# Endoplasmic Reticulum Thiol Oxidase Deficiency Leads to Ascorbic Acid Depletion and Noncanonical Scurvy in Mice

**DOI:** 10.1016/j.molcel.2012.08.010

**Published:** 2012-10-12

**Authors:** Ester Zito, Henning Gram Hansen, Giles S.H. Yeo, Junichi Fujii, David Ron

**Affiliations:** 1 University of Cambridge Metabolic Research Laboratories and NIHR Cambridge Biomedical Research Centre, Cambridge CB2 0QQ, UK; 2 Department of Biochemistry and Molecular Biology, Graduate School of Medical Science, Yamagata University, Yamagata 990-9585, Japan

## Abstract

Endoplasmic reticulum (ER) thiol oxidases initiate a disulfide relay to oxidatively fold secreted proteins. We found that combined loss-of-function mutations in genes encoding the ER thiol oxidases ERO1α, ERO1β, and PRDX4 compromised the extracellular matrix in mice and interfered with the intracellular maturation of procollagen. These severe abnormalities were associated with an unexpectedly modest delay in disulfide bond formation in secreted proteins but a profound, 5-fold lower procollagen 4-hydroxyproline content and enhanced cysteinyl sulfenic acid modification of ER proteins. Tissue ascorbic acid content was lower in mutant mice, and ascorbic acid supplementation improved procollagen maturation and lowered sulfenic acid content in vivo. In vitro, the presence of a sulfenic acid donor accelerated the oxidative inactivation of ascorbate by an H_2_O_2_-generating system. Compromised ER disulfide relay thus exposes protein thiols to competing oxidation to sulfenic acid, resulting in depletion of ascorbic acid, impaired procollagen proline 4-hydroxylation, and a noncanonical form of scurvy.

## Graphical abstract

**Graphical Abstract:**

**Figure:**
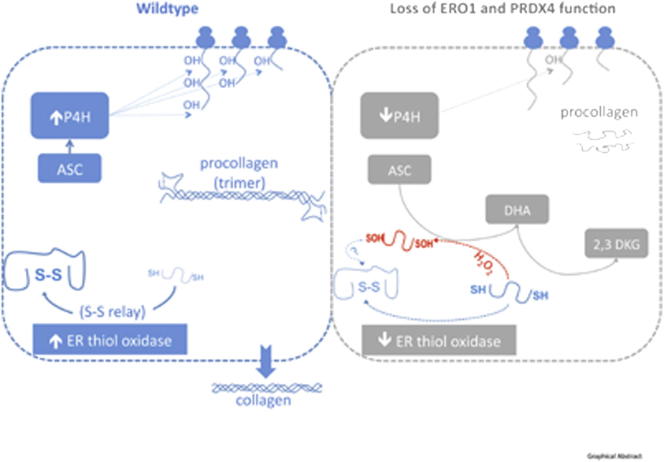

**Highlights:**

► Abnormal extracellular matrix in mice compromised in their ER thiol oxidases ► Abnormal procollagen maturation responsive to ascorbate repletion ► Tissue ascorbate depletion and noncanonical scurvy in the mutant mice

## Introduction

Disulfide bonds have a critical role in stabilizing the mature, folded conformation of secreted proteins (Anfinsen, 1973). A specialized class of chaperones of the protein disulfide isomerase (PDI) family catalyze efficient formation of disulfides in folding ER proteins. Oxidized PDIs transfer their disulfides onto reduced ER client proteins and are themselves reduced in this terminal step of disulfide relay (Ellgaard and Ruddock, 2005; Hatahet and Ruddock, 2009). Upstream in this relay the ER is endowed with enzymes of the ER oxidoreducin 1 (ERO1) family (Margittai and Sitia, 2011; Sevier and Kaiser, 2008; Tu and Weissman, 2004) and, in higher eukaryotes, with a peroxiredoxin (PRDX4) (Tavender et al., 2008; Zito et al., 2010b) that couples the reoxidation of PDI thiols to the reduction of nonthiol electron acceptors, such as molecular oxygen or hydrogen peroxide.

The cellular function of the ER oxidases has been studied most extensively in yeast, where deletion of the single *ERO1* gene results in death due to failure of oxidative protein folding (Frand and Kaiser, 1998; Pollard et al., 1998). Mammals have two ERO1 isoforms, ERO1α and ERO1β (encoded by the *Ero1l* and *Ero1lb* genes in mice) (Cabibbo et al., 2000; Pagani et al., 2000), and thiol oxidation is further backed up by PRDX4 (Tavender and Bulleid, 2010; Zito et al., 2010b). Loss-of-function mutations in mammalian ERO1 genes result in rather subtle phenotypes. Mice lacking ERO1β develop a mild nonprogressive pancreatic β cell dysfunction with glucose intolerance, and mice lacking ERO1α have an abnormal cardiac response to adrenergic stimulation (Chin et al., 2011; Zito et al., 2010a). *Prdx4* null mice have a subtle defect in spermatogenesis but no reported defect in metabolism of ER proteins (Iuchi et al., 2009).

The aforementioned relatively minor phenotypes correlate with the subtlety of the kinetic defect in disulfide bond formation in the ERO1-compromised mammals. Thus, it seemed reasonable to assume that the dramatically different outcome of interfering with ERO1 function in yeast and mammals reflects the existence of redundant mechanisms for thiol oxidation in the latter and that a sufficiently severe kinetic defect in the ER disulfide relay would register first and foremost as a failure of oxidative protein folding in mammals too. This simple assumption is supported by the dramatic increase in ER unfolded protein stress signaling in mammalian cells treated with agents, like dithiothreitol (DTT), that reduce disulfide bonds (Brostrom et al., 1995).

Multiple mechanisms have been proposed to account for the apparent redundancy of pathways to disulfide bond formation in mammals. These include other ER thiol oxidases (Nguyen et al., 2011; Thorpe and Kodali, 2010) and nonenzymatic processes (Ruddock, 2012). The latter result in the production of chemical species not normally elaborated during the PDI-mediated disulfide relay initiated by the enzymatic thiol oxidases. This brings up an interesting theoretical point: the kinetic-dominated view of ER thiol chemistry emphasizes the rate at which disulfides form between protein thiols and has focused on the destabilizing effect of delays in this process on protein folding. However, kinetic defects in the enzymatically mediated disulfide relay are also predicted to predispose the expanded pool of free ER protein thiols to competing nonenzymatic oxidation and thus promote an expanded repertoire of chemical species, beyond thiols and disulfides.

One pathway for such nonenzymatic oxidation entails the formation of sulfenic acid by the direct reaction of cysteine thiols with H_2_O_2_. This outcome would be favored by elimination of enzymes, like PRDX4, that scavenge H_2_O_2_ in the ER (Poole et al., 2011). A sulfenic acid may interact with a free thiol, forming a disulfide and bypassing the protein-based disulfide relay (Rehder and Borges, 2011). But sulfenic acid also has the potential to interact with other electron donors. For example, ascorbic acid can reduce protein sulfenic acids back to a free thiol, as has been shown to occur in the active site of one-cysteine peroxiredoxin (Monteiro et al., 2007). During the process, ascorbic acid is converted to dehydroascorbate, which is chemically unstable. Thus, theoretical considerations suggest that altered kinetics of disulfide bond formation may affect aspects of ER function beyond the rate of oxidative protein folding, leading, for example, to depletion of lumenal ascorbic acid content.

Here we report on the characterization of mice with impaired activity of the enzymes ERO1 and PRDX4 that initiate the disulfide relay. Our observations point to an unanticipated defect in intracellular ascorbic acid metabolism that culminates in a scorbutic extracellular matrix.

## Results

### Disulfide Bond Formation Proceeds in T^M^ Cells Lacking Three ER Thiol Oxidases

To examine the consequences of combined deficiency of both mammalian ERO1 enzymes and PRDX4, we mated female mice heterozygous for the previously described *Ero1l* mutant allele, *Ero1l*^*Gt(XST171)Byg*^ (referred to here as *Ero1α*^*m*^) (Chin et al., 2011), homozygous for the previously described *Ero1lb* mutant allele, *Ero1lb*^*Gt(P077G11)Wrst*^ (referred to here as *Ero1β*^*m*^) (Zito et al., 2010a) and heterozygous for the previously described X-linked *Prdx4* null allele, *Prdx4*^*tm1Jufu*^ (referred to here as *Prdx4*^*m*^) (Iuchi et al., 2009), to male *Ero1α*^*m/m*^*; Ero1β*^*m/m*^ mice (referred to here as double mutant, D^M^ mice). Previously we had observed that D^M^ mouse embryonic fibroblasts (MEFs) were intolerant of compromised *Prdx4* expression, reflected in the severe relative fitness cost associated with acquisition of shRNA lentiviruses targeting the *Prdx4* gene in the D^M^ background compared with the wild-type background (Zito et al., 2010b). These observations, reflecting a genetic redundancy in the function of ERO1 and PRDX4 in MEFs, led us to expect a severe early embryonic phenotype of *Ero1α*^*m/m*^; *Ero1β*^*m/m*^; *Prdx4*^*m/y*^ triple mutant (T^M^) individuals, due to failure of oxidative protein folding. Surprisingly, however, rare individuals with the triple mutant genotype were recovered, albeit with lower frequency than that predicted by Mendelian transmission of the mutant alleles (Table 1).

**Figure 1:**
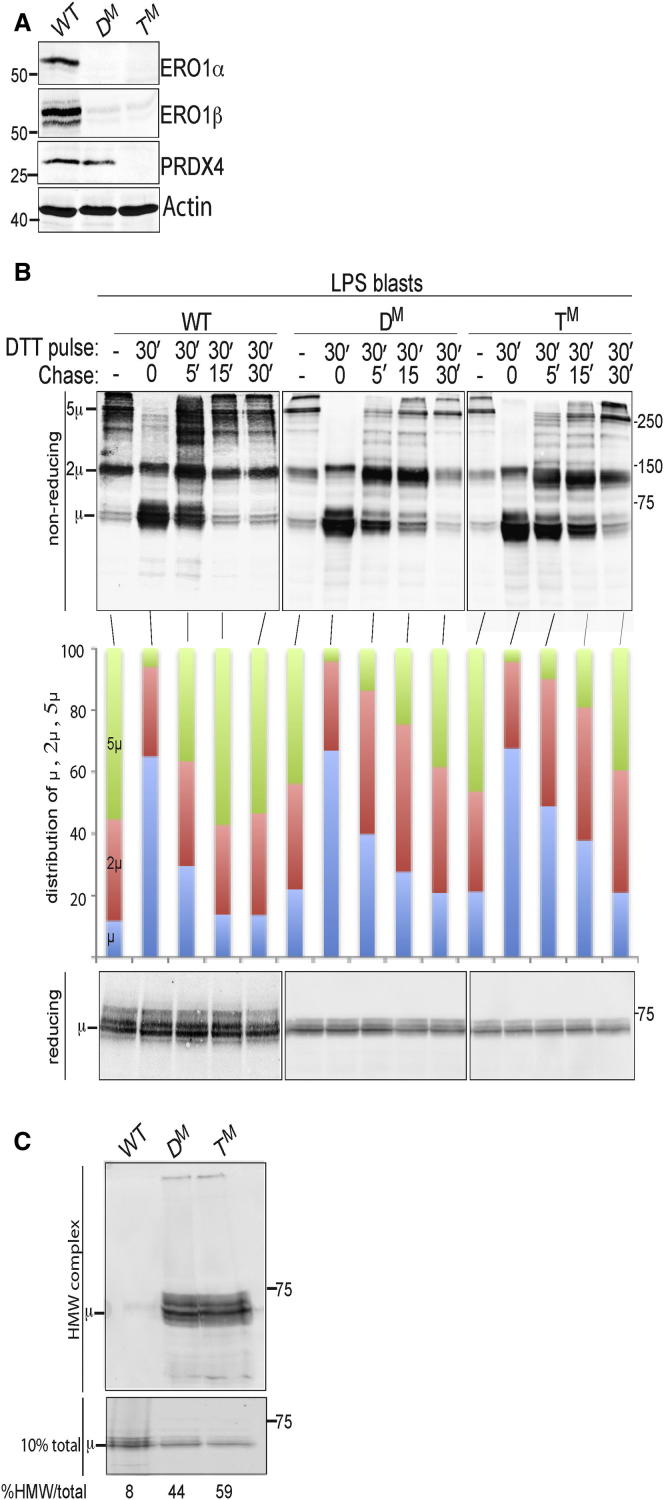
A Subtle Kinetic Defect in Disulfide Bond Formation in Mice Compromised in ERO1 and PRDX4 (A) Immunoblot of the indicated proteins in extracts of lipopolysaccharide-induced lymphoblasts produced from wild-type (WT), *Ero1α*^*m/m*^; *Ero1β*^*m/m*^ compound double mutant (D^M^), and *Ero1α*^*m/m*^; *Ero1β*^*m/m*^; *Prdx4*^*m/y*^ compound triple mutant (T^M^) mice. (B) Nonreducing and reducing immunoblot of IgM heavy chain extracted from lipopolysaccharide-induced lymphoblasts of the indicated genotypes. Where noted, the cells were exposed to a 30 min pulse of the reducing agent dithiothreitol (DTT, 10 mM) followed by a washout period. The migration of IgM monomers (μ), dimers (2μ), and pentamers (5μ) is noted, and their relative distribution in the various samples is depicted graphically in the central panel. Note that despite the minor kinetic defect, nearly all the reduced monomeric IgM is converted to oxidized higher-molecular-weight forms in the mutant cells over time. (C) Reducing immunoblot of insoluble high-molecular-weight complex (HMW) and total IgM heavy chain extracted from lipopolysaccharide-induced lymphoblasts of the indicated genotypes. The HMW complex (upper panel) was recovered as a pellet from the detergent lysates layered on a glycerol cushion, as previously described (Marciniak et al., 2004). The lower panel reports of the total content of IgM heavy chain in the sample and the relative content of insoluble HMW complex is noted below each lane.

To assess the impact of the triple mutant genotype on rate of disulfide bond formation in the ER of secretory cells, we prepared lipopolysaccharide stimulated splenocytes (so-called LPS blasts) from T^M^, D^M^, and wild-type animals and compared the rate at which disulfide bonds recovered in the cell-associated pool of immunoglobulin M (IgM) following a pulse of the reducing agent DTT. While a subtle kinetic delay was evident in both mutant samples (and was slightly more conspicuous in the T^M^ compared with the D^M^ cells), this experiment showed that disulfide bond formation proceeded rapidly even in cells deficient in all three ER thiol oxidases, ERO1α, ERO1β, and PRDX4 (Figures 1A and 1B).

Despite the subtlety of the kinetic delay in disulfide bond reformation, defective ER function was clearly evident in both D^M^ and T^M^ cells. The fraction of cell-associated IgM recovered in high-molecular-weight SDS-insoluble complexes (Marciniak et al., 2004) was 5- and 7-fold greater in the D^M^ and T^M^ cells compared to the wild-type (Figure 1C).

ERO1β is highly enriched in pancreatic β cells, and its dysfunction compromises insulin secretion. Previously, we observed that the mild diabetic phenotype of the *Ero1β*^*m/m*^ mice is unaffected by further compromise of *Ero1α* (Zito et al., 2010a), and here we noted that it is only minimally worse in compound *Ero1β*^*m/m*^; *Prdx4*^*m/y*^ mice (see Figure S1 online). These observations further attest to the redundancy in pathways to disulfide bond formation and presented two interesting questions: What is the basis of the redundancy of the ERO1s and PRDX4 as upstream thiol oxidases? And, given the subtlety of the kinetic defect in disulfide formation, what accounts for the strong fitness cost of the T^M^ genotype? This study is focused on the latter question.

### Defective Connective Tissue in Mice Lacking ER Thiol Oxidases

**Figure 2:**
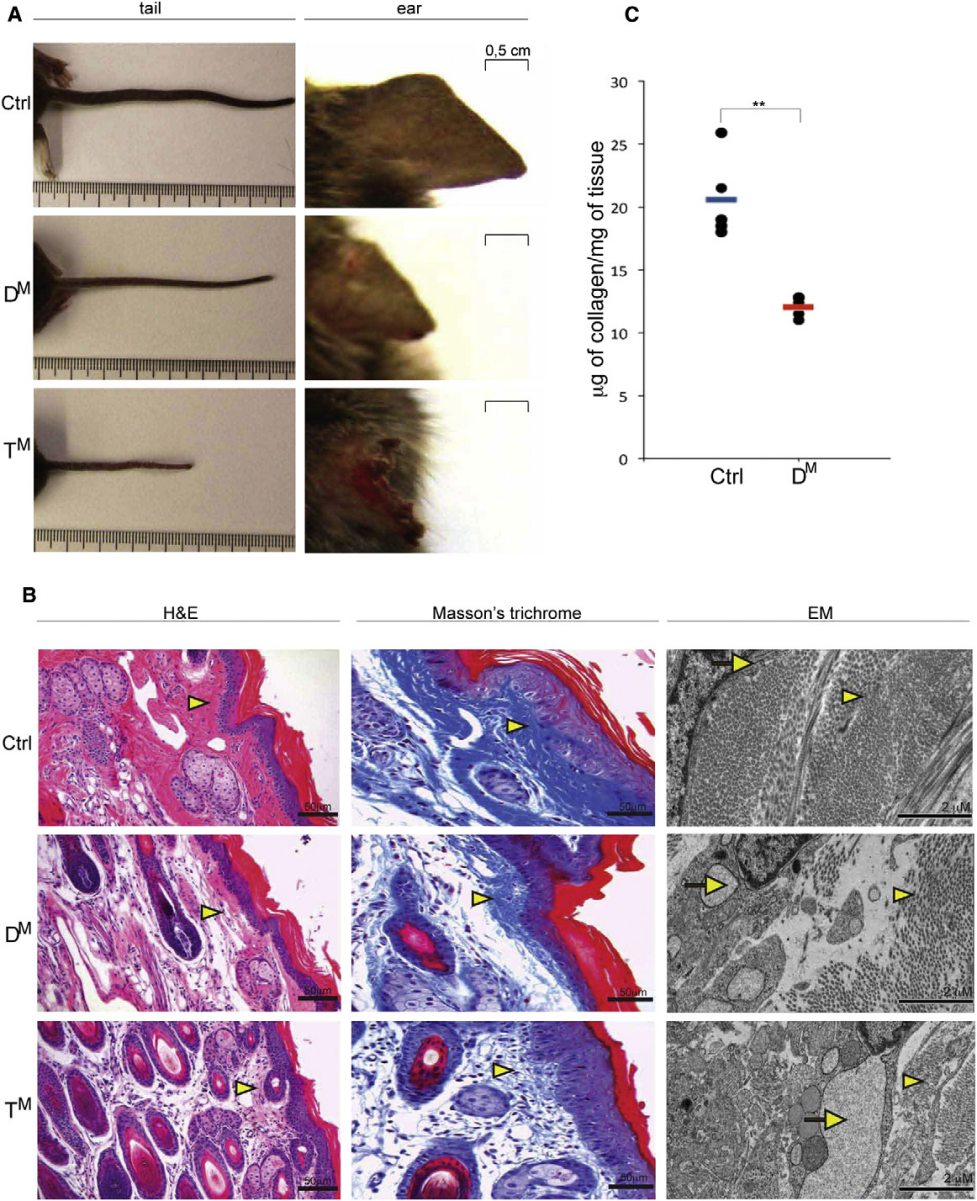
Abnormal Connective Tissue in the D^M^ and T^M^ Mice (A) Photomicrographs of the tails and ears of 8-week-old control (Ctrl, as defined in Table 1), D^M^, and T^M^ littermates. Note the thin and misshapen tail and degenerated pinna of the T^M^ and the abnormally thin but normally proportioned tail and smaller pinna of the D^M^ mice. (B) Hematoxylin and eosin (H&E)-stained, Masson’s Trichrome-stained paraffin embedded sections and electron micrographs (EM) of skin from tail of mice of the indicated genotype. Note the abnormally sparse connective tissue fibers in the dermis of the mutant samples (yellow arrowheads in the H&E- and Masson’s Trichrome-stained sections, more conspicuous in the T^M^ than the D^M^ sample) and the dilated ER (arrow) in tissue fibroblasts and abnormal connective tissue fibers (arrowhead) in the mutant EM samples. (C) Collagen mass (measured as 4-hydroxyproline content in an acid hydrolysate of skin, normalized to a standard mass of purified rat tail collagen) per tissue mass recovered from mice of the indicated genotypes. Shown are values obtained in individual samples and the mean of each group (n = 4, ^∗∗^p < 0.01).

T^M^ animals had a conspicuous phenotype: they were less than 70% in weight of their littermates, failed to thrive, and their tails and ears were disproportionately small and misshapen and, in rare survivors, degenerated with time (Figure 2A). Closer scrutiny revealed that D^M^ animals, too, had thin tails and smaller ears, an invariable phenotype that was previously overlooked (Chin et al., 2011).

Histological analysis of the tail showed markedly less fibrous eosinophilic material in the dermis of the D^M^ and even less in the T^M^ mice. The presence of abnormal connective tissue fibers in the tissue of the mutants was confirmed by Masson’s Trichrome Blue staining. Electron microscopy showed dilated ER in fibroblasts in the mutant tissue and abnormally small, loosely packed, and disorganized fibrils, which were especially conspicuous in the T^M^ sample (Figure 2B). Levels of tissue 4-hydroxy proline (a measure of collagen content) were >40% lower in the D^M^ tail tissue compared to the control genotype (Figure 2C; we were unable to recover enough mutant animals to obtain meaningful measurements of this parameter in the T^M^ genotype).

**Figure 3:**
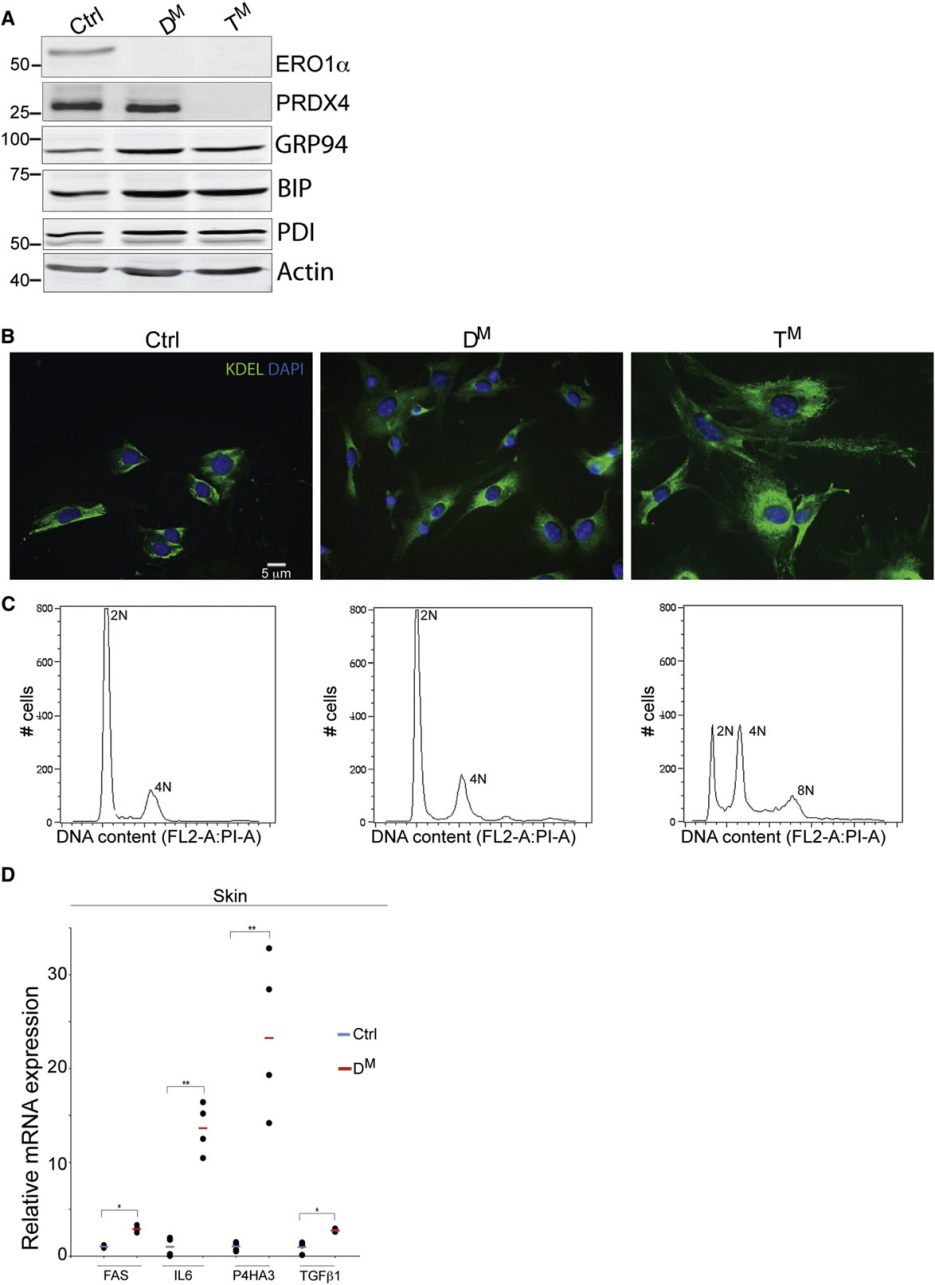
Elevated TGF-β Signature in Mutant MEFs and Mouse Tissue (A) Immunoblot of ERO1α, PRDX4, GRP94, BiP, PDI, and Actin in extracts of primary MEFs from embryos of the indicated genotypes. (B) Photomicrographs of fixed primary MEFs of the indicated genotype stained with an antibody to the KDEL-COOH peptide (to delineate the ER) and DAPI to outline the nucleus. Note the flattened morphology and enlarged nuclei of the T^M^ cells. (C) Fluorescence-activated cell scanning (FACS) profiles of MEFs stained with the DNA-binding dye, propidium iodide. Note the prominent population of T^M^ cells with abnormally high DNA content. (D) Relative abundance of TGF-β pathway mRNAs measured by quantitative real-time PCR in cDNA from skin. Shown are the values obtained in individual mice and the mean for each genotype (n = 4, ^∗^p < 0.05, ^∗∗^p < 0.01).

The aforementioned observations indicated that the connective tissue of mice with compromised ER thiol oxidases was defective. To explore this problem, we turned to MEFs, a cell culture model for connective tissue. Immunoblot confirmed the presence of ERO1α and PRDX4 in the control population (ERO1β is not detectable in MEFs, Zito et al., 2010a) and their absence in the D^M^ and T^M^ populations. Levels of the ER chaperones BiP and GRP94 were only minimally elevated in the mutant cells, indicating comparable levels of unfolded protein stress in the ER of MEFs of the three genotypes (Figure 3A).

Despite the absence of markers of excessive ER stress, the T^M^ MEFs were distinguishable in appearance: they were larger and flatter than the control (or D^M^) MEFs, with conspicuously larger nuclei (Figure 3B). Fluorescence-activated cell scanning (FACS) following staining with the DNA-binding dye, propidium iodide, revealed a prominent population of T^M^ cells with abnormally high DNA content, with an apparent modal distribution consistent with 2N, 4N, and 8N cells (Figure 3C and Figure S2A).

### An Elevated TGF-β Signature and Impaired Procollagen Maturation in Cells Compromised in ER Thiol Oxidases

The abnormal cellular morphology and DNA content suggested the presence of a substantial fraction of senescent cells in the pool of T^M^ MEFs. To explore this further, we analyzed the profile of mRNAs expressed by MEFs of the three genotypes by gene expression microarrays. Pathway analysis revealed hyperactivity of the interconnected transforming growth factor β (TGF-β), and the P38 mitogen-activated protein kinase (P38 MAPK) pathways in the D^M^ and T^M^ cells (Figures S2B–S2E). Importantly, a subset of these target genes were also elevated in mRNA samples procured from the skin of D^M^ mice (Figure 3D).

The elevated TGF-β signature, with an attendant increase in IL-6 and P38 MAPK activity, nicely explains the growth-inhibited (Assoian and Sporn, 1986) and senescent phenotype (Rodier and Campisi, 2011) of the mutant cells. As altered TGF-β signaling often accompanies defects in the extracellular matrix (Ramirez and Rifkin, 2009), and as such defects were evident in the mutant mice (Figure 2B), we turned our attention to collagen, a major extracellular matrix protein produced by MEFs.

**Figure 4:**
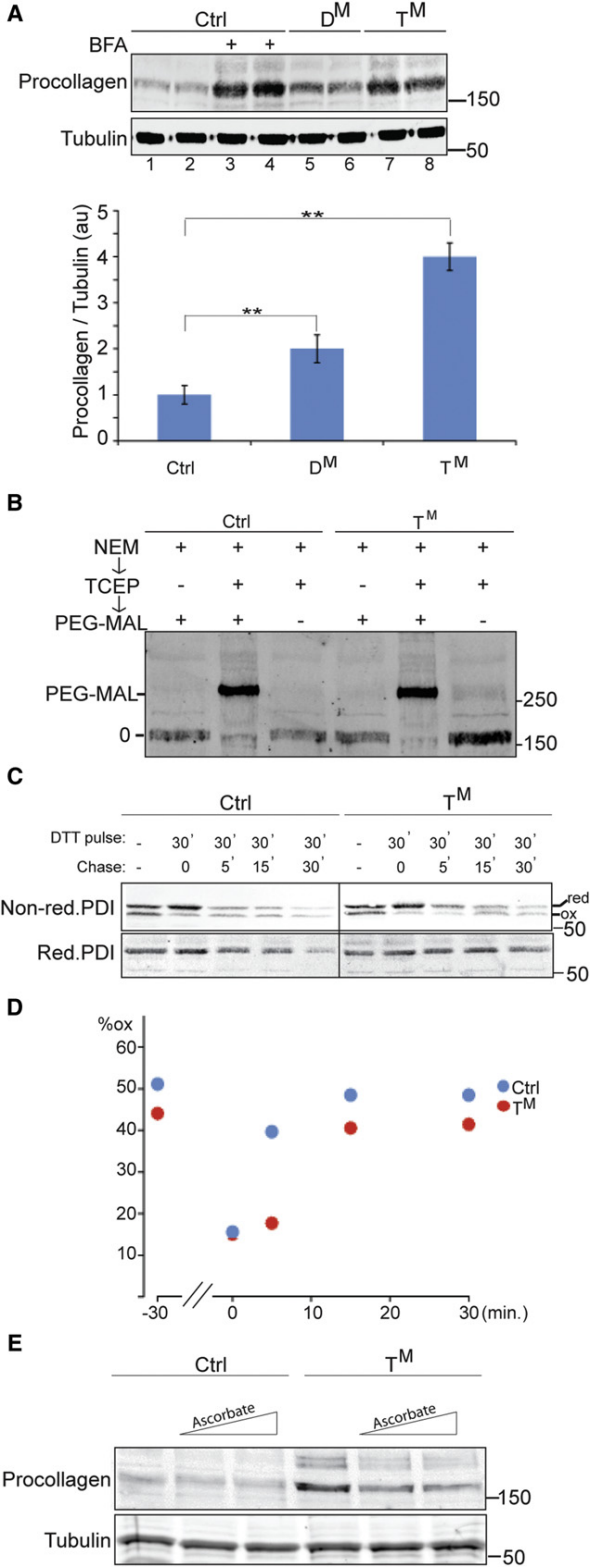
An Ascorbic Acid-Responsive Defect in Type I Procollagen Maturation in Mutant Cells (A) Immunoblot of type I procollagen in lysates of MEFs of the indicated genotypes. Where noted, cells were exposed to brefeldin A (BFA, 2 μg/mL) for 3 hr before harvest to inhibit export of procollagen from the ER. The anti-Tubulin blot (lower panel) serves a loading control. Shown is a representative experiment performed in duplicate. The bar diagram below the immunoblot shows the mean ± SEM of the ratio of the procollagen to tubulin signal expressed in arbitrary units (au) from three such experiments (n = 3, ^∗∗^p < 0.01). (B) Immunoblot of procollagen from BFA-treated MEFs. Where indicated, disulfides in the sample were reduced with TCEP before exposure to the thiol-modifying agent PEG-MAL-2000. The modified (PEG-MAL) and unmodified (0) species are marked. (C) Nonreducing and reducing immunoblot of protein disulfide isomerase (PDI) following exposure of MEFs to the reducing agent dithiothreitol (DTT pulse) and a washout for the indicated time (chase). The migration of reduced and oxidized forms of PDI is noted. (D) Plot of oxidized PDI as percentage of the total from the experiment shown in (C). Note the subtlety of the kinetic defect in PDI reoxidation following DTT washout in the mutant cells. (E) Procollagen immunoblot, as in (A). Where indicated, the culture media was supplemented with ascorbate (to a concentration of 50 μM or 100 μM).

Type I collagen, the abundant species made by MEFs, is synthesized as a precursor, procollagen. The precursor is modified cotranslationally by lumenal proline and lysine hydroxylases, and later trimer-stabilizing disulfide bonds form between the C-terminal propieces. The procollagen trimer traffics rapidly from the ER and is efficiently processed to mature collagen in post-ER compartments (Lamande and Bateman, 1999). Therefore wild-type MEFs have low levels of procollagen. Exposure to brefeldin A (BFA), which interferes with ER trafficking, traps procollagen in the ER and increases its steady-state levels (Figure 4A, lanes 1–4). Both D^M^ and T^M^ MEFs have abnormally elevated steady-state levels of procollagen (Figure 4A). These observations point to a defect in procollagen maturation in the mutant cells.

To explore the disulfide bonds in procollagen, wild-type and T^M^ MEFS were treated with BFA to trap procollagen in their ER. Free thiols were quenched with N-ethyl maleimide (NEM), and proteins were precipitated with trichloroacetate (TCA). Following removal of the NEM from the pellet, disulfide bonds were reduced with phosphine and the newly exposed free thiols alkylated with NEM conjugated to polyethylene glycol, resulting in a size shift of the protein’s mobility on SDS-PAGE that is proportional to number of disulfides that had been present before reduction. The large mass of procollagen precluded a precise count of such disulfides, but the similar size shift in procollagen argued against a conspicuous defect in disulfide bonding in the T^M^ MEFS (Figure 4B). Moreover, comparison of the rate of disulfide bond formation in the ER of wild-type and T^M^ MEFs, by tracking the recovery, following a reducing pulse of DTT, of a higher mobility, oxidized PDI isoform (recognized on nonreducing immunoblots by the SPA-891 antibody from Stressgen, Chin et al., 2011) shows that, like the T^M^ lipopolysaccharide blasts, the T^M^ fibroblasts had merely a subtle delay in disulfide bond reformation on this sentinel PDI (Figures 4C and 4D). These findings argued against a major kinetic defect in disulfide bond formation that could explain the retention of procollagen in the mutant cells.

As slow oxidative folding seemed inadequate to account for procollagen accumulation in the ER of mutant MEFs, we sought alternative explanations. A clue was provided by the observation that supplementing the growth media of the mutant cells with ascorbic acid partially reverses the retention of procollagen (Figure 4E).

### An Ascorbate-Responsive Defect in Proline 4 Hydroxylation in Cells Lacking ER Thiol Oxidases

**Figure 5:**
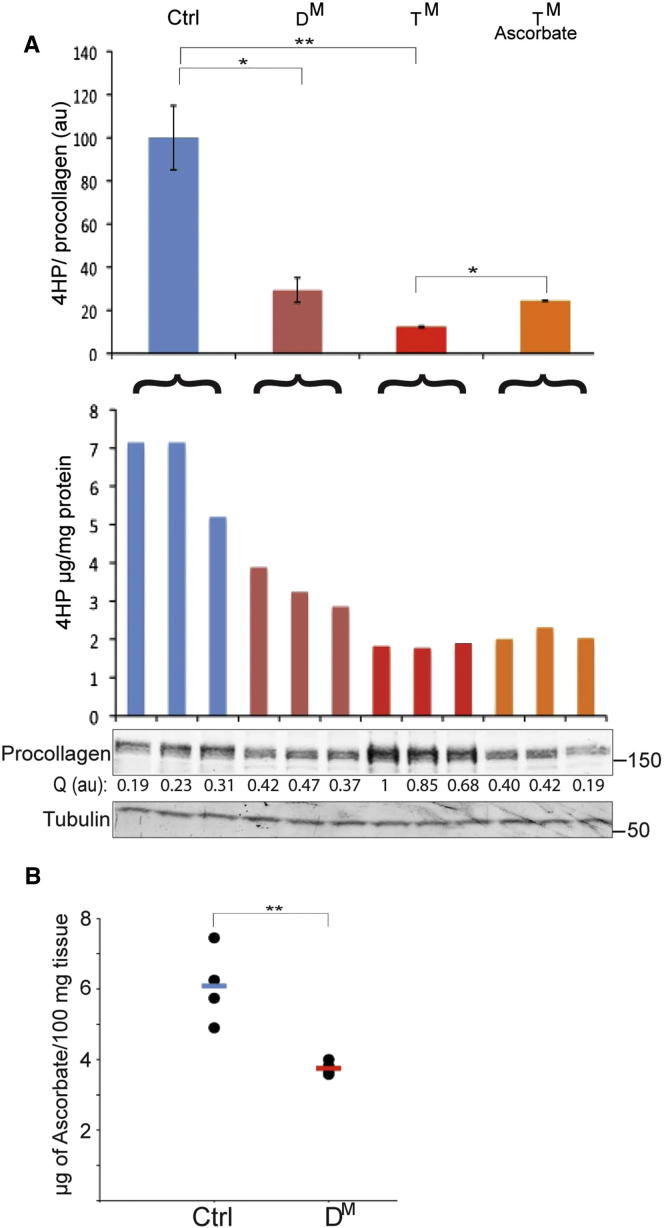
Impaired Proline 4 Hydroxylation of Procollagen in Mutant Fibroblasts (A) 4-hydroxyproline content of lysates of brefeldin A-treated MEFs, normalized to the procollagen content of the cells. The upper bar diagram shows the mean ± SEM of the normalized values, expressed in arbitrary units (au) (n = 3, ^∗∗^p < 0.001, ^∗^p < 0.05). The lower bar diagram depicts the 4-hydroxyproline content in the individual samples. The procollagen content of each sample was quantified from the fluorescent signal of the immunoblot and is presented under the procollagen blot (Q) in arbitrary units (au). (B) Ascorbic acid content of skin of the indicated mice. Shown are the values obtained in individual animals and the mean for each group (n = 4, ^∗∗^p < 0.01).

Ascorbic acid maintains the activity of ER-localized proline 4-hydroxylases. Failure of proline 4 hydroxylation blocks procollagen secretion and leads to its retention in the ER (Juva et al., 1966). To determine if the procollagen retained in the ER of the mutant cells had abnormally low levels of 4-hydroxyproline, we used BFA to trap procollagen in the ER of MEFs of all three genotypes and assayed the cellular content of 4-hydroxyproline by hydrolysing proteins to amino acids and converting 4-hydroxyproline to a pyrrole by sequential oxidation and decarboxylation, in a colorigenic reaction (Prockop and Udenfriend, 1960). To validate this approach to trapping procollagen in wild-type cells, we confirmed that the 4-hydroxyproline content of wild-type MEFs increased following BFA treatment (Figure S3A). Procollagen 4-hydroxyproline content was 3-fold higher in the BFA-treated wild-type MEFs compared to the D^M^ and 6-fold higher than in the T^M^ cells (Figure 5A).

Proline 4-hydroxylation is a cotranslation process that precedes the formation of critical interchain disulfides (Wilson et al., 1998). Therefore, the defect observed in the mutant cells was not readily explained by slower rates of disulfide bond formation in collagen, leading us to consider alternative explanations. Adequate levels of ascorbic acid are required to maintain the catalytic iron atom of proline 4-hydroxylases in the active +2 redox state (Peterkofsky and Udenfriend, 1965). Failure of this enzyme-preserving process results in scurvy, a disease characterized by impaired secretion of extracellular matrix proteins (Wolbach and Bessey, 1942). Interestingly, addition of ascorbic acid to the culture media resulted in a 2-fold increase in 4-hydroxyproline content of procollagen trapped by BFA in the T^M^ cells (Figure 5A) and commensurate lower steady-state levels of procollagen in untreated mutant cells (Figure 4E).

The responsiveness of mutant cells to supplementation suggested a functional deficiency in ascorbate. This defect correlated with 1.6-fold lower ascorbate content in the tissue of the D^M^ mice (3.75 ± 0.2 μg ascorbate/100 mg tissue) compared with littermate controls (6.04 ± 1.1 μg ascorbate/100 mg tissue) (Figure 5B). The lower tissue content of ascorbate in the mutant is consistent with the defect in proline 4-hydroxylation and suggests that ascorbate may be preferentially depleted from the mutant cells.

**Figure 6:**
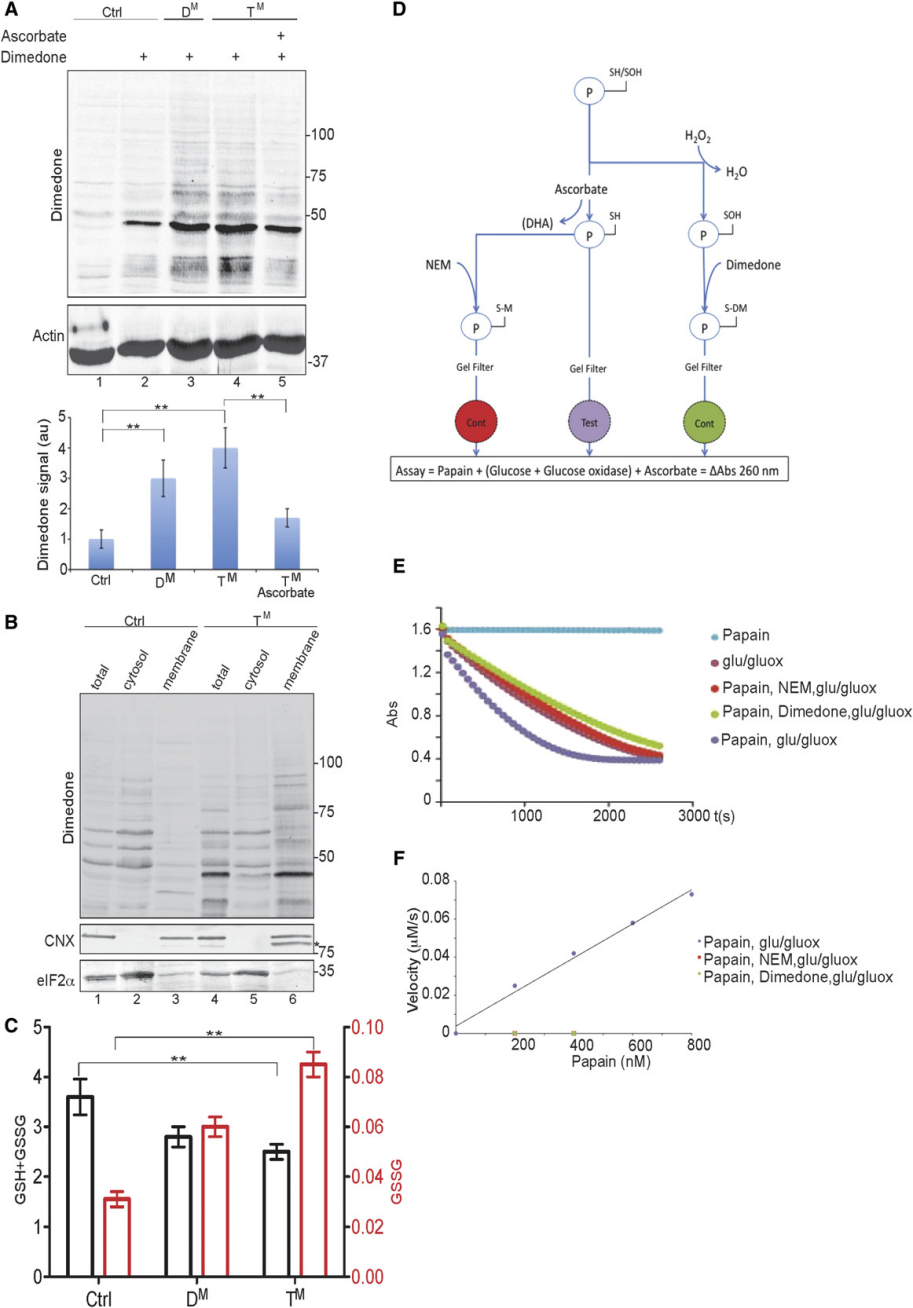
Elevated Cysteinyl Sulfenic Acid in Proteins from Mutant Cells and Their Role in Ascorbate Depletion (A) Representative immunoblot of extracts from cells exposed to the sulfenic acid reactive probe dimedone with an antibody reactive to dimedone-conjugated cysteine residues. The bar diagram is the mean ± SEM of the dimedone signal (integrated across each lane and expressed in arbitrary units, au) from experiments as shown above (n = 3, ^∗∗^p < 0.01). (B) As in (A), but the cells were fractionated to a cytosolic fraction and a membrane (microsomal) fraction by digitonin permeabilization and cytosolic squeeze-out. Calnexin (CNX) and eIF2α serve as compartment markers for the ER and cytosol. The asterisk indicates a faster-migrating species reactive with the calnexin antiserum that is reproducibly detected in the membrane fraction of the mutant cells and may correspond to sulfenylated calnexin (Leonard et al., 2009). (C) Bar diagram of the total glutathione (GSH + GSSG) and oxidized glutathione (GSSG) content in picomols glutathione/μg of proteins of cells of the indicated genotypes (n = 3, ^∗∗^p < 0.01). (D) Schema of the experiment to test the effect of protein cysteinyl sulfenic acid in catalyzing ascorbic acid oxidation. The active site cysteine of papain (P) is found as both a thiol (−SH) and cysteinyl sulfenic acid (−SOH, due to air oxidation). H_2_O_2_ converts all the −SH to −SOH, which then reacts irreversibly with dimedone, blocking the active cysteine (−S−DM). Following gel filtration, to remove remaining H_2_O_2_ and dimedone, this generates an inactive control (Cont). Ascorbate reduces any −SOH back to −SH. Removal of the ascorbate by gel filtration generates the active (Test) protein. However, the free thiol can be blocked by NEM, generating an inactive alkylated (−S−M) protein (Cont). The test and control proteins are incorporated at substochiometric concentrations into an assay with an H_2_O_2_-generating system (glucose + glucose oxidase) and ascorbate, whose consumption is measured by reading the absorbance at 265 nm. (E) Time-dependent changes in absorbance of ascorbate (at 265 nm, initial concentration 100 μM) observed in the experimental system described in (B). “Papain,” trace of sample with no H_2_O_2_-generating system but with 0.2 μM papain. “glu/gluox,” trace of sample with no papain. “Papain, NEM, glu/gluox,” trace of sample with NEM-blocked papain (0.2 μM). “Papain, Dimedone, glu/gluox,” trace of sample with dimedone-blocked papain (0.2 μM). “Papain, glu/gluox,” trace of sample with active papain (0.2 μM). (F) The initial rate of ascorbate oxidation (in OD units/time) as a function of papain concentration (the initial rate of the uncatalyzed reaction, with only glucose and glucose oxidase, was subtracted).

Ascorbic acid can react with cysteinyl sulfenic acid side chains. The resulting transfer of two electrons converts ascorbate to an unstable oxidized derivative, dehydroascorbate (which is rapidly hydrolysed to 2,3 diketo l-gulonate), and reduces the sulfenic acid, regenerating the free thiol (Monteiro et al., 2007). Therefore, elevated levels of sulfenylated cysteines could accelerate the clearance of ascorbate from the mutant cell’s ER and contribute to the defect in collagen biogenesis. To compare the burden of sulfenylated proteins in MEFs of divergent genotypes, we exposed cells and lysates to dimedone, a chemical that selectively modifies sulfenylated cysteines, and detected the dimedone-modified proteins by immunoblot with an antibody to the chemically modified group (Seo and Carroll, 2009). In absence of dimedone, the antibody gave a weak background signal (Figure 6A, lane 1), and the dimedone-dependent signal was effaced by incubating cells with DTT, a direct reductant of sulfenic acid (Figure S4). Importantly, the sulfenic acid signal was notably higher in the D^M^ and T^M^ cells (Figure 6A, lanes 3 and 4) and was enriched in the ER-containing membrane fraction (Figure 6B). The quantity of sulfenylated proteins detected by dimedone modification decreased following exposure to ascorbate (Figure 6A, lane 5), an observation consistent with a role for ascorbate as a reductant of sulfenylated proteins.

An elevated ratio of oxidized to total glutathione has been previously noted in the D^M^ MEFs (Appenzeller-Herzog et al., 2010) and in HepG2 cells subjected to ERO1 and PRDX4 inactivation by shRNA (Rutkevich and Williams, 2012). These findings were confirmed in the D^M^ and T^M^ MEFs (Figure 6C), suggesting that lower levels of a competing reductant (reduced glutathione) may contribute to sulfenylated protein-mediated oxidation of ascorbate.

To directly measure the potential of sulfenylated proteins to serve as ascorbate oxidants, we compared the rate of decay of ascorbate in an in vitro reaction consisting of glucose and glucose oxidase (a source of H_2_O_2_ which serves as an ultimate electron acceptor) alone:

$$H_{2}O_{2}+Ascorbate\to H_{2}O+Dehydroascorbate$$

or in the presence of papain, an enzyme whose active site thiol is rapidly oxidized to a sulfenic acid in the presence of H_2_O_2_ (Klomsiri et al., 2010; Sluyterman, 1967):

$$\begin{array}{l} H_{2}O_{2}+papain^{SH}\to H_{2}O+papain^{SOH}\\ papain^{SOH}+Ascorbate\to papain^{SH}+Dehydroascorbate. \end{array}$$

To control for nonspecific effects of papain on the rate of ascorbate oxidation, the active site cysteine was blocked by dimedone (after its conversion to a sulfenic acid) or by NEM (after its reduction to a thiol) (Figure 6D).

In the papain-catalyzed coupled reaction, the absorbance signal from ascorbate declined more rapidly than in the uncatalyzed reaction. This acceleration by papain was dependent on the integrity of papain’s free thiol, as blocking the reduced thiol with NEM or the oxidized (sulfenylated) cysteine with dimedone (Figure 6D) abolished the papain-mediated acceleration of ascorbate depletion (Figure 6E).

Papain-mediated acceleration of ascorbate oxidation was evident at papain:ascorbate molar ratios as low as 1:500 and increased with increased papain concentration (Figure 6F), demonstrating that papain is working catalytically in this reaction. Unfortunately, impurities in the papain preparation that inhibited the reaction at protein concentrations greater than 1 μM precluded detailed study of the reaction kinetics. Nonetheless, these observations showcase the potential for sulfenic acid to deplete ascorbate while leaving open the question of the impact of competing reductants in the ER. The higher levels of sulfenylated proteins in the mutant cells, which decreased upon ascorbate treatment, together with the in vitro demonstration of accelerated ascorbate oxidation by a protein sulfenic acid, suggest a mechanism for ascorbate depletion in the mutant cells.

## Discussion

This study confirmed in mice observations made earlier in cultured cells on the redundancy between the ERO1 enzymes (ERO1α and ERO1β) and PRDX4 in initiating a disulfide relay in the ER of mammals. However, contrary to expectation, the kinetic defect in oxidative protein folding induced by mutation in these three upstream ER thiol oxidases was rather modest. Instead, the phenotype of the mutant mice and the biochemical characterization of their cells and tissues revealed an unanticipated consequence of altered ER thiol redox kinetics on intracellular ascorbate metabolism.

Free cysteine thiols that are not converted quickly enough to disulfides are exposed to an alternative fate: oxidation to sulfenic acid. Reduction of the excess cysteinyl-sulfenic acid side chains back to the free thiol converts ascorbate to an unstable oxidized derivative. The subtle kinetic defect in disulfide bond formation depletes the mutant ER of an essential factor (vitamin C), promoting an unconventional form of scurvy with profound defects in the extracellular matrix. This study therefore provides a remarkable example of the emerging links between altered ER protein metabolism and intermediary metabolism.

ERO1 and PRDX4 are biochemically distinct enzymes, but they have in common the ability to initiate a disulfide relay in the ER (Tavender and Bulleid, 2010; Zito et al., 2010b). *Prdx4* mutant mice and single gene defects in the *Ero1* isoforms have no evident abnormality in the extracellular matrix. The defect is first apparent in the compound *Ero1α*^*m/m*^*; Ero1β*^*m/m*^ D^M^ mice and is dramatic in triply compromised *Ero1α*^*m/m*^*; Ero1β*^*m/m*^*; Prdx4*^*m/y*^ T^M^ mice, providing formal genetic evidence that the three ER thiol oxidases have partially redundant roles. The progressive compromise of the extracellular matrix with compounding of mutations is mirrored by the progressive retention of procollagen and in the progressive increase in levels of cysteinyl-sulfenic acid in mutant cultured fibroblasts. These genotype-phenotype correlations thus argue that a kinetic defect in disulfide bond formation, common to the three mutations, underlies the aforementioned abnormalities in ER function.

Disulfide formation and oxidation to sulfenic acid are alternative fates of cysteinyl thiols in the ER lumen. The combined defect in disulfide relay and in the conversion of H_2_O_2_ (to water and a disulfide) synergistically biases the competition in favor of cysteinyl thiols in the T^M^ cells and readily explains the strong dimedone reactivity of protein samples from T^M^ cells. However, the accumulation of cysteinyl thiols in the D^M^ cells (which have no known defect in ER peroxidase activity) and the absence of such a defect in the PRDX4 mutant cells, which lack a peroxidase, suggest that merely a kinetic delay in disulfide formation is sufficient to expose free thiols to this alternative fate.

The transfer of two electrons from a free thiol to peroxide produces cysteinyl sulfenic acid. ERO1 generates H_2_O_2_ in course of disulfide bond formation (Gross et al., 2006). However, both the previously noted redundancy of ERO1 and PRDX4 (Zito et al., 2010b; Rutkevich and Williams, 2012) and the abundance of cysteinyl sulfenic acid in the ER of D^M^ cells, noted here, exonerate ERO1 as a major contributor to ER peroxide content in mammals; alternative sources, such as mitochondrial respiration and ER-localized NADPH oxidase(s), may dominate the production of peroxide.

Formation of cysteinyl sulfenic acid on one thiol followed by its resolution by a second thiol creates a disulfide (and a water molecule). In the presence of reduced PDI, the disulfide can isomerise to a correctly placed protein disulfide, or it can be reduced back to the dithiol, initiating a PDI-mediated disulfide relay (Karala et al., 2009). However, both processes are slow, and the cysteinyl sulfenic acid, an intermediate in the pathway to disulfide bond formation, is subject to alternative fates. Our observations suggest that by providing an efficient cysteinyl sulfenic acid-independent route to disulfide bond formation, the ERO1- and PRDX4-initiated disulfide relay pre-empts this alternative. Thus, the ER of the mutant cells is not paradoxically hyperoxidizing but rather coherently misoxidizing.

Ascorbate is the active ingredient in vitamin C, whose absence from the diet results in human scurvy. Mice are able to synthesize ascorbate in their liver and thus do not require a dietary source of the vitamin. Ascorbate is taken up by cells, most likely as dehydroascorbate, which is rapidly converted back to ascorbate in the reducing environment of the cytosol (Linster and Van Schaftingen, 2007). It is unclear how the active vitamin (ascorbate) makes its way into the ER lumen to exert its antiscorbutic effect. But our findings argue that, once in the ER, ascorbate can reduce cysteinyl-sulfenic acid side chains of ER proteins. The two electrons transferred in this process oxidize ascorbate to dehydroascorbate (Monteiro et al., 2007). The dehydroascorbate could, in theory, reoxidize thiols to disulfides, contributing to the ER disulfide relay, but this process is too slow for effective recycling back to ascorbate (Saaranen et al., 2010). In plants, reduced glutathione can be utilized to enzymatically generate ascorbate from dehydroascorbate, in the first step of the Halliwell-Asada cycle (reviewed in Noctor and Foyer, 1998). However, it is unclear if such enzymatic activity is conserved in animal cells and, if so, whether it is present in the ER. Thus the pool of reduced GSH may well be kinetically isolated from dehydroascorbate produced in the ER and the competing nonenzymatic process, whereby dehydroascorbate hydrolyzed to 2,3-diketo-l-gulonate (2,3-DKG) likely predominates (Bode et al., 1990). Unlike dehydroascorbate, which has some antiscorbutic activity (because it can be reduced to ascorbate), 2,3-DKG is a dead-end product, and its formation is a net loss of vitamin C.

The cysteinyl-sulfenic acid-driven consumption of ER ascorbate proposed above parallels a similar accelerated depletion of ascorbate reported in cells exposed to Ni^+2^ or Co^+2^ (Salnikow et al., 2004). The parallels extend further, as heavy-metal-mediated ascorbate depletion occurs against a background of high concentration of reduced glutathione and also culminates in the inactivation of the cytoplasmic proline 4 hydroxylases, the PHD enzymes charged with oxygen sensing (Kaczmarek et al., 2007). While our experiments outline the feasibility of direct depletion of ascorbate by excessive cysteinyl-sulfenic acid side chains of ER client proteins, we cannot exclude the contribution of other radicals generated in the mutant cells. Furthermore, abnormally high oxidized to reduced glutathione ratios have been recently noted in cells compromised in ERO1 (Appenzeller-Herzog et al., 2010; Rutkevich and Williams, 2012) and were confirmed here and suggest that depletion of reduced glutathione might favor a situation whereby nascent proteins, oxidized to sulfenic species, selectively exploit ascorbate as their reductant in the mutant cells. This issue also affects the interpretation of the in vitro demonstration of cysteinyl-sulfenic acid-driven consumption of ascorbate (Figure 6E), which, because of the uncertainty surrounding the concentration of reduced glutathione in the ER, does not take into account the potential for a competing reduction pathway.

The defect in collagen biosynthesis and the abnormal extracellular collagen fibrils are associated with abnormalities of the skin of the ER oxidase-compromised mice, a conspicuous feature of which is an elevated TGF-β gene expression signature and elevated levels of the inflammatory cytokine IL-6. These observations are consistent with a role for the extracellular matrix in regulating TGF-β signaling (Ramirez and Rifkin, 2009). They are also consistent with the developing notion that altered TGF-β signaling may contribute to the pleiotrophy of primary genetic defects in extracellular matrix proteins (Lindsay and Dietz, 2011).

The chain of events linking perturbed ER redox to tissue level abnormalities in extracellular matrix was exposed here through targeted mutagenesis of ER thiol oxidases. However, ER stress, associated with delayed egress of client proteins from the ER and excessive oxidative stress (Malhotra et al., 2008), accompanies other pathophysiological states and is especially prominent in the context of the metabolic syndrome of obesity and cardiovascular disease (Hotamisligil, 2010). It is thus tempting to speculate on the possibility that intracellular ascorbate depletion by its reaction with cysteinyl sulfenic acid may starve some cells of vitamin C and thus contribute to altered secretion of extracellular proteins and tissue-level abnormalities in a range of disease states.

## Experimental Procedures

### Animal Experiments

Animal breeding and experiments were approved by the University of Cambridge Bioethics Committee.

### Analysis of Disulfides in Procollagen

MEFs were alkylated with NEM and lysed in 10% TCA. TCA pellets were washed and resuspended in 0.5 M Tris HCl (pH 7.5), 5% SDS. Some of the samples were reduced with TCEP (tris [2-carboxyethyl] phosphine hydrochloride) (Sigma) and free thiols alkylated with PEG-maleimide 2000 (SUNBRIGHT ME-020MA, NOF Europe). The proteins were resolved on a reducing SDS-PAGE.

### 4-Hydroxyproline Content

4-hydroxyproline content of cell or tissue acid hydrolysate was measured by the method of Prokop and Udenfriend (Prockop and Udenfriend, 1960) using the QuickZyme Total collagen assay kit (QuickZyme BioSciences, Netherlands) according to the manufacturer’s instructions.

### Detecting Protein Sulfenic Acids in Cultured Cells

Sulfenic acid-modified proteins were detected following the procedure of Seo and colleagues (Seo and Carroll, 2009). Briefly, cells were trypsinized, pelleted (200 × g at room temperature), and resuspended in low-protein media (1% FCS) containing 10 mM dimedone (Acros Organics) or DMSO (2.5% vol/vol) and incubated for 2 hr at 37°C, followed by lysis in an ice-cold dimedone- and catalase-containing buffer and incubated for 30 min on ice with frequent mixing. Sulfenic acid-modified proteins were detected by immunoblot using a rabbit serum reactive to sulphenic acid modified proteins (Millipore, catalog number AbS30).

Where indicated, MEFs were fractionated into a cytosolic and membrane fraction by digitonin permeabilization followed by cytoplasmic squeeze out (Shamu et al., 1999).

### Sulphenylated Protein-Mediated Decay of Ascorbic Acid In Vitro

Papain from Carica papaya (Sigma) was equilibrated to 100 mM sodium phosphate (pH 7.0) via PD-10 desalting column (GE Healthcare). To obtain dimedone-blocked papain, a solution of papain (50 μM in phosphate buffer) was exposed to H_2_O_2_ (50 μM) and dimedone (1 mM) for 30 min at room temperature and gel filtered again. To obtain NEM-blocked papain, papain was exposed ascorbate (100 μM) for 30 min followed by NEM (1 mM) and gel filtered again. Active or blocked papain (at concentrations of 0.2–0.8 μM) was combined with 100 μM of ascorbic acid in the presence of glucose (2.5 mM) and glucose oxidase (type II from *Aspergillus niger*, Sigma, 10 mU/ml) in the same phosphate buffer, and the decay of ascorbic acid was measured by monitoring the decline in absorbance at 265 nm.

### Ascorbic Acid and Glutathione Content

Skin from control and mutant animals was homogenized in 60% methanol containing 0.05% EDTA. The proteins and insoluble material were clarified by centrifugation. The supernatant was dried and ascorbic acid estimated with a colorimetric assay as described (Jagota and Dani, 1982).

Cellular levels of total and oxidized glutathione were measured fluorometrically using the DTNB glutathione reductase recycling assay as described previously (Griffith, 1980).

## Figures and Tables

**Table 1:** Detrimental Effect of the PRDX4 Loss-of-Function Mutation in *Ero1α^m/m^; Ero1β^m/m^* Mice

Description	Genotype			Sex	Number		Percentage of Total	
	*Ero1α*	*Ero1β*	*Prdx4*		Ex	Ob	Ex	Ob
Control	m/+	m/m	+/+	F	16.1	23	12.5	17.8
Control	m/+	m/m	m/+	F	16.1	24	12.5	18.6
D^M^	m/m	m/m	+/+	F	16.1	20	12.5	15.5
D^M^ <=> T^M^^∗^	m/m	m/m	m/+	F	16.1	6	12.5	4.65
Control	m/+	m/m	+/y	M	16.1	20	12.5	15.5
Control	m/+	m/m	m/y	M	16.1	19	12.5	14.7
D^M^	m/m	m/m	+/y	M	16.1	14	12.5	10.9
T^M^	m/m	m/m	m/y	M	16.1	3	12.5	2.3
					Total	129		

The expected (Ex) and observed (Ob) distribution of genotypes at weaning among 129 progeny of a cross between *Ero1α*^*m/+*^; *Ero1β*^*m/m*^; *Prdx4*^*m/+*^ females and *Ero1α*^*m/m*^; *Ero1β*^*m/m*^; *Prdx4*^*+/y*^ males (χ-square test = 26.47 with seven degrees of freedom, p = 0.0004). ^∗^Due to X chromosome inactivation, female *Ero1α*^*m/m*^; *Ero1β*^*m/m*^; *Prdx4*^*m/+*^X are somatic mosaics constituted of double-mutant (D^M^) and triple-mutant (T^M^) cells; the standard notation of chimerism, “<=>,” is used to indicate this.

## Accession Numbers

The GEO accession number of the microarray data is GSE39492.

## Supplemental Information

Document S1. Figure S1, Figure S2, Figure S3, Figure S4, Supplemental Experimental Procedures, and Supplemental References
