# Supplementary material for: Endoplasmic Reticulum Thiol Oxidase Deficiency Leads to Ascorbic Acid Depletion and Noncanonical Scurvy in Mice
Source: Mol Cell. 2012 Oct 12;48(1):39–51. doi: 10.1016/j.molcel.2012.08.010 (PMC3473360; doi:10.1016/j.molcel.2012.08.010)
Supplement: Document S1. Figure S1, Figure S2, Figure S3, Figure S4, Supplemental Experimental Procedures, and Supplemental References [file mmc1.pdf]

Molecular Cell, Volume *48*

Supplemental Information

Endoplasmic Reticulum Thiol Oxidase Deficiency Leads to Ascorbic Acid Depletion and Noncanonical Scurvy in Mice

Ester Zito, Henning Gram Hansen, Giles S.H. Yeo, Junichi Fujii, and David Ron

This supplemental section contains 4 supplemental figures and Supplemental Experimental Procedures.

Figure S1 relates to the main Figure 1 reports on the mildly enhanced diabetic phenotype of mice with PRDX4 deficiency compounding ERO1 deficiency

Figure S2 relates to the main Figure 3 reports on the cell-cycle and gene expression defects associated with compound deficiency of ERO1 and PRDX4

Figure S3 relates to the main Figure 5 reports on the kinetics of brefeldin-A mediated retention of procollagen

Figure S4 relates to the main Figure 6 reports on the sensitivity the dimedone reactivity of proteins in T^M cells to reduction by DTT

The Supplemental Experimental Procedures section describes the methods used in animal experiments, measurements of immunoglobulin oxidation in lipopolysaccharide blasts, isolation of control, D^M and T^M mutant MEFs, analysis of the cell cycle and gene expression.

The Supplemental References pertain to the Supplemental Experimental Procedures.

Figure S1

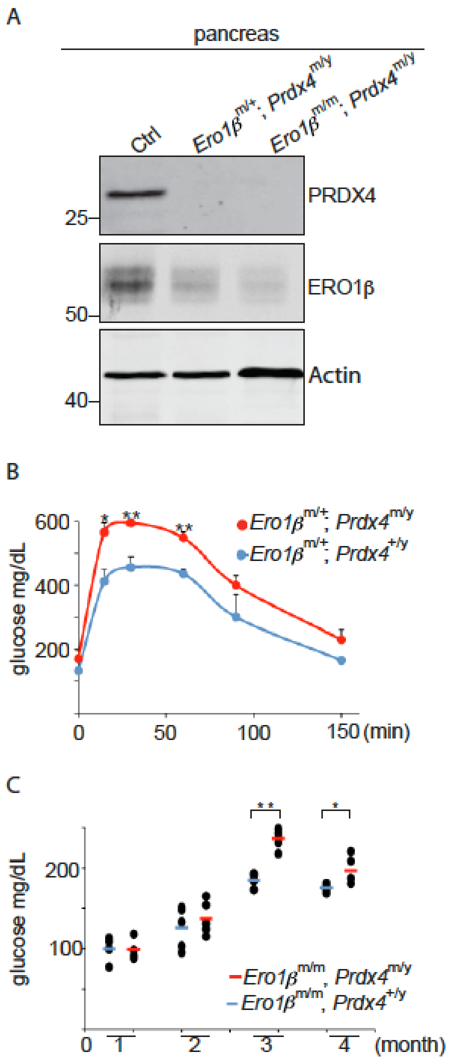

Figure S1 (related to main Figure 1):

A. Immunoblot of ERO1β, PRDX4 and Actin in pancreatic lysates of the indicated genotypes.

B. Blood glucose levels following an intra-peritoneal injection of glucose (2 mg/ gm body weight) in 8 week old *Ero1β*$^{m/+}$ and *Ero1β*$^{m/+}$; *Prdx4*$^{m/y}$ male siblings. Shown are mean ± SEM (n=5 **p<0.01, *p<0.05). Note the modest glucose intolerance imposed by the compounding *Prdx4* deletion.

C. Fasting blood glucose of *Ero1β*$^{m/m}$ and compound *Ero1β*$^{m/m}$; *Prdx4*$^{m/y}$ male siblings of the indicated age. Shown are the values obtained in individual animals and the mean for each group (n=5 **p<0.01, *p<0.05). The mild, stable, fasting hyperglycemia, previously noted in the *Ero1β*$^{m/m}$ mice is only mildly increased by the compounding *Prdx4* deletion.

Figure S2

Figure S2 (related to the main Figure 3):

A. FACS profiles of untreated MEFs and MEFs following exposure to the ER stress-inducing toxin tunicamycin (2.5 µg/ml for 16 hours) stained with the DNA-binding dye, propidium iodide. Note the presence of a hypo-diploid population in the treated samples of both genotypes, validating the 2N, 4N and polyploidy peaks of the mutant.

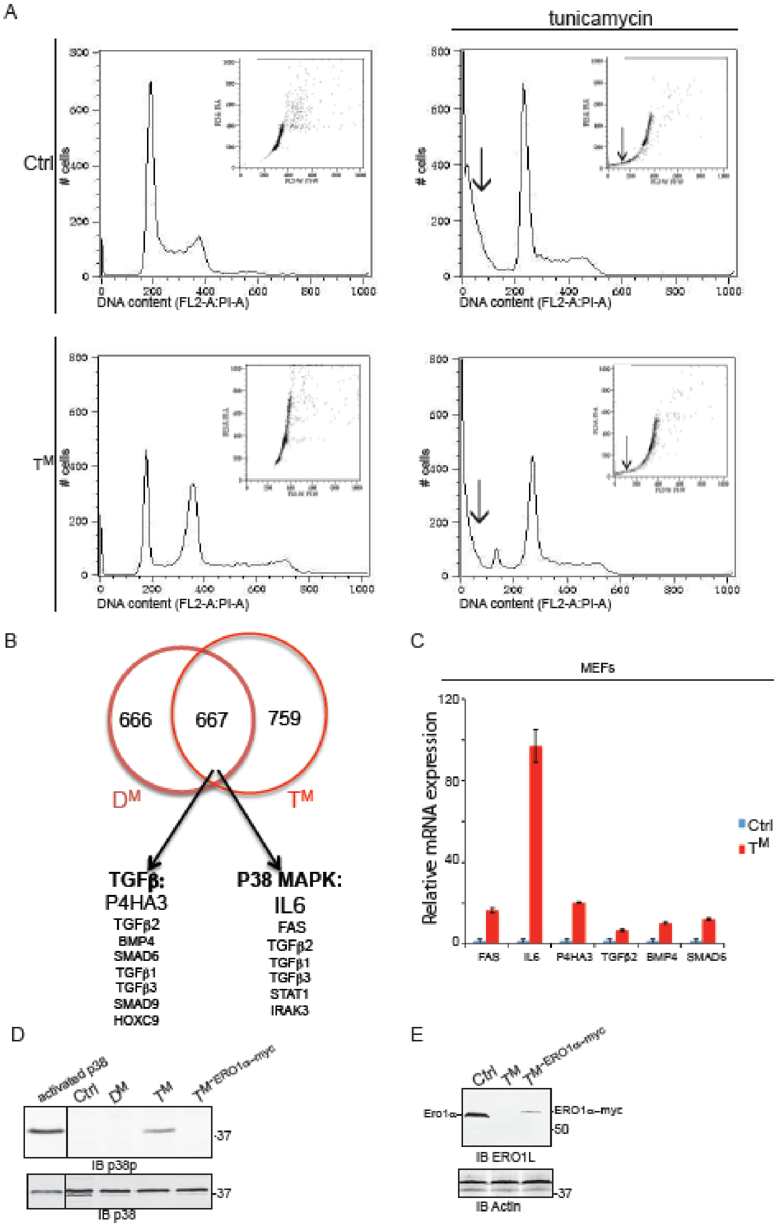

B. Ven diagram showing the overlap of genes whose expression is 1.5 fold higher in the D^M and T^M MEFs compared to the reference genotype. Genes annotated as belonging to the TGFβ and P38 MAP kinase pathway and found in the overlap group are listed (the font size is proportional to the over-expression in the T^M sample).

C. Relative abundance of TGFβ pathway mRNAs measured by real time quantitative-PCR in extracts of MEFs of the indicated genotype.

D. Immunoblot of phosphorylated (activated) P38 MAP kinase in extracts from MEFs of the indicated genotype. The positive control was from wildtype MEFs serum starved for 48 hours. Note the lack of an activated P38 signal in the TM cells transduced with a rescuing ERO1α transgene.

E. Immunoblot of ERO1α documenting the rescue of its expression in the indicated clone.

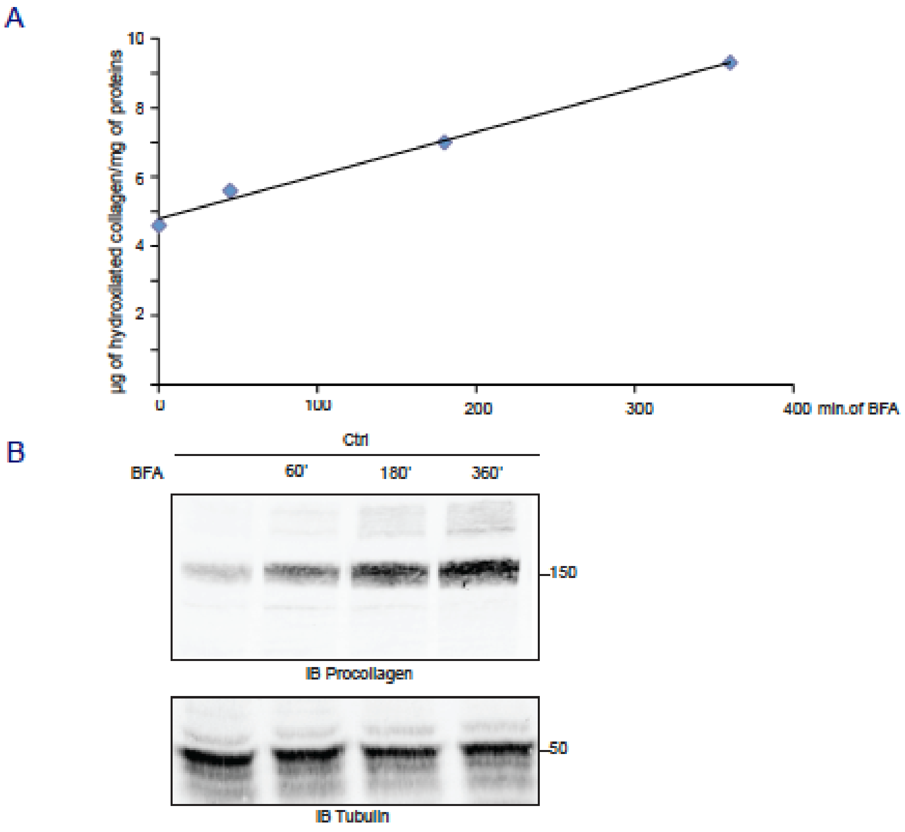

Figure S3 (related to the main Figure 5):

A. The 4 hydroxyproline content of wildtype MEFS following exposure to Brefeldin A.

B. Procollagen immunoblot of the cells shown in “A”

Figure S4

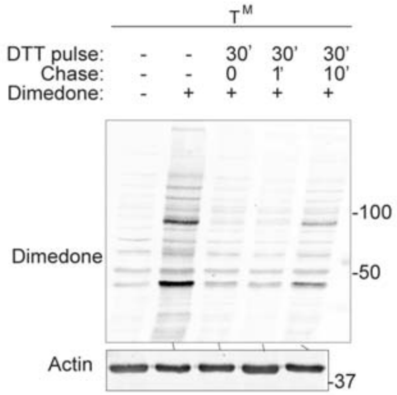

Figure S4 (related to the main Figure 6):

Immunoblot of T^{M} cell extracts with an antibody reactive to dimedone-conjugated cysteine residues. The cells were exposed for 30 minutes in vivo to the reducing agent DTT (10 mM) following a washout period of the indicated time. Note the elimination of the baseline signal with the DTT pulse and its return during the washout (chase period).

Supplemental Experimental Procedures

Animal experiments

Animal breeding and experiments were approved by the University of Cambridge Bioethics committee and the UK Home Office. Genotyping at the *Ero1l*, *Ero1lb* and *Prdx4* loci followed published procedures (Iuchi et al., 2009; Zito et al., 2010a). Skin from age-matched control and mutant animals was fixed in formalin. Masson's trichrome and H&E staining was carried out as described (Bradshaw et al., 2003). For electron microscopy samples were fixed by immersion in 2.5% glutaraldehyde in 0.1 M Sorensen buffer, postfixed in 1% osmium tetroxide, and en bloc stained with 3% uranyl acetate. Ultrathin sections were poststained with uranyl acetate and lead citrate and examined by electron microscopy as described (Zito et al., 2010a).

Isolation, culture and immunoglobulin oxidation in lipopolysaccharide blasts.

A previously-published procedure was followed (Zito et al., 2010a, figure 6C). Briefly, cells teased from disrupted spleens of mice with wildtype and compound D^M and T^M genotypes were cultured in RPMI-1640 at a density of 10^6 cells/ml and exposed to 50 µg/ml lipopolysaccharide from *E. Coli* (Sigma, L2755) for 2-3 days. Following a 30 min pulse with 10 mM DTT, chase was performed in complete media followed by lysis in the presence of N-ethyl maleimide (NEM, 20 mM), non-reducing or reducing SDS-PAGE and immunoblotting with rabbit anti-mouse IgM (Rockland, 610-4107). Fractionation of the IgM into low and high molecular weight complex was performed by challenging the NP-40 solubilized lysates with 0.5% SDS and centrifugation through a glycerol cushion, as described (Marciniak et al., 2004).

Cytoplasmic squeeze-out

MEFs were fractionated into a cytosolic and membrane fraction by digitonin permeabilization followed by cytoplasmic squeeze-out (Shamu et al., 1999). Briefly, following incubated on ice with PBS containing 40 µg/ml-1 digitonin for 10 minutes. Cells were scraped off the plate, the cytosol extruded as a supernatant by centrifugation at maximum speed in a table top centrifuges for 10 minutes. The pellet was extracted in 1% triton X-100 to yield the detergent soluble proteins from the membrane fraction. Both fractions were analyzed by SDS-PAGE followed by Immunoblot assay.

Isolation and analysis of control, D^M and T^M mutant MEFs

Control ($Ero1\alpha^{m/+}$; $Ero1\beta^{m/m}$; $Prdx4^{+/+}$), D^M ($Ero1\alpha^{m/m}$; $Ero1\beta^{m/m}$; $Prdx4^{+/+}$) and T^M ($Ero1\alpha^{m/m}$; $Ero1\beta^{m/m}$; $Prdx4^{m/y}$) mutant mouse embryonic fibroblasts (MEFs), isolated at embryonic day 13.5, were studied as I˚ MEFs (figure 3 and S3) or immortalized with SV-40 large T antigen (Figure 4-6) were cultured in DMEM supplemented to 25 mM glucose, 10% FCS, non essential amino acids and where indicated, were exposed to Brefeldin A (2 µg/mL), L-ascorbic acid (100 µM) or tunicamycin (2.5 µg/ml) (Sigma).

Immunoblots were developed with far red fluorescent II˚ antisera on a LiCor Odyssey and the signal quantified using Image Studio V2.0. The following I˚ immunochemical reagents were used: Rabbit anti-ERO1α and ERO1β (Zito et al., 2010a) PRDX4 (Zito et al., 2010b), anti-PDI (Stressgen, SPA-891, Figure 4B), anti-KDEL (Stressgen, SPA-827D, Figure 3A and 3B), anti–□-actin (Sigma A5441) and tubulin (Sigma T9026), anti-P38 (total Cell Signaling Technology 9212L and phosphorylated 9211L), anti-type I mouse collagen (Rockland, 600-401-103).

Microarray analysis

Each experiment comprised triplicate RNA samples of MEFs of the three genotypes. Labeled cRNA was hybridized to Affimetrix Gene ST1.0 chips.Raw image data were converted to CEL files using Affymetrix GeneChip Operating Software (GCOS). All downstream analysis of microarray data was performed using GeneSpring GX 11.0 (Agilent). Data were analysed using both Robust Multiarray Average (RMA) and Probe Logarithmic Intensity Error (PLIER) algorithms. Genes were considered differentially regulated at greater than 1.5-fold change ($p<0.05$) as compared to WT MEFs. Only genes that met the above criteria using both RMA and PLIER were taken forward for additional study. Pathway analyses were performed using Ingenuity Pathway Analysis (IPA) (Ingenuity Systems Inc., www.ingenuity.com).

Real-time quantitative RT-PCR analysis

Skin from adult mice (5 month old) and MEFs were homogenized in Trizol lysis reagent, following the manufacturer's instructions. Poly-dT primed reverse transcription (RT) was carried out using RevertAid Premium Reverse Transcriptase (Thermo Fischer Scientic, Waltham, MA) according to the manufacturer's protocol. Real-time quantitative RT-PCR reactions were run on an ABI Prism 7000 Sequence Detection System using the qPCR Core Kit for SYBR Green PCR Master Mix (Life Technologies, Paisley, UK) and analyzed using ABI Prism 7000 SDS Software (Applied Biosystems, Foster City, CA). Relative mRNA levels of all genes were first normalized to the levels of β-Actin and Cyclophilin-A using the 2(-Delta Delta C (T)) method then normalized to the average of control levels. The primer sequences for the transcripts quantified by this method are shown below.

**Primer mM**	**Sense (5'-3')**	**Antisense (5'-3')**
Ppia	TTCCTCCTTTCACAGAATTATTCA	CCGCCAGTGCCATTATGG
Bmp4	TCAAGGGAGTGGAGATTGGG	GCCATCATGGCCAAAAGTG
β–Actin	GTATGGAATCCTGTGGCATC	AAGCACTTGCGGTGCACGAT
Fasn	GCA AACCAGACTTCTACTGCGA	TTTGTATTGCTGGTTGCTGTGC
IL6	AAAGAGTTGTGCAATGGCAATTCT	AAGTGCATCATCGTTGTTCATACA
P4ha3	TTGACCATGCTACGTCGCCCAG	AAAGCCGTGGCTCCTCCAGCTT
Smad6	TGGCTGGAGATCCTACTCAACA	GGACGCTGCGGCACAG
Tgfb1	AGCCCGAAGCGGACTACTAT	TTCCACATGTTGCTCCACAC
Tgfb2	GCGAAGAGCTCGAGGCGAGAT	GAGAATGGTCAGTGGTTCCAGAT

Cell Cycle Analysis by FACS

MEFs were fixed in 90% ethanol overnight at -20˚C, permeabilized with 0.5% NP-40 and briefly treated with RNase A (final concentration of 250 µg/ml), stained with propidium iodide (10 µg/ml, 37˚C for 5 min) and signal intensity measured on a FACScan benchtop flow cytometer (Becton Dickinson, San Jose, CA). Ten thousand events were acquired and analyzed with CellQuest software (Becton Dickinson).

Statistics

All results are expressed as means ± SEM. Two-tailed Student's t tests were performed to determine p values for paired samples was performed for the experiments that employed more than one independent variable. χ-square statistical analysis was performed on the distribution of genotypes of Table1.

Supplemental References

Bradshaw, A.D., Puolakkainen, P., Dasgupta, J., Davidson, J.M., Wight, T.N., and Helene Sage, E. (2003). SPARC-null mice display abnormalities in the dermis characterized by decreased collagen fibril diameter and reduced tensile strength. J. Invest. Dermatol. *120*, 949-955.
